# Carbonic anhydrase III (Car3) is not required for fatty acid synthesis and does not protect against high-fat diet induced obesity in mice

**DOI:** 10.1371/journal.pone.0176502

**Published:** 2017-04-24

**Authors:** Sarah W. Renner, Lauren M. Walker, Lawrence J. Forsberg, Jonathan Z. Sexton, Jay E. Brenman

**Affiliations:** 1 Genetics and Molecular Biology Curriculum, University of North Carolina at Chapel Hill, Chapel Hill, North Carolina, United States of America; 2 UNC Neuroscience Center, University of North Carolina at Chapel Hill, Chapel Hill, North Carolina, United States of America; 3 Biomanufacturing Research Institute and Technology Enterprise, North Carolina Central University, Durham, North Carolina, United States of America; 4 Department of Cell Biology and Physiology, University of North Carolina at Chapel Hill, Chapel Hill, North Carolina, United States of America; INRA, FRANCE

## Abstract

Carbonic anhydrases are a family of enzymes that catalyze the reversible condensation of water and carbon dioxide to carbonic acid, which spontaneously dissociates to bicarbonate. Carbonic anhydrase III (Car3) is nutritionally regulated at both the mRNA and protein level. It is highly enriched in tissues that synthesize and/or store fat: liver, white adipose tissue, brown adipose tissue, and skeletal muscle. Previous characterization of Car3 knockout mice focused on mice fed standard diets, not high-fat diets that significantly alter the tissues that highly express Car3. We observed lower protein levels of Car3 in high-fat diet fed mice treated with niclosamide, a drug published to improve fatty liver symptoms in mice. However, it is unknown if Car3 is simply a biomarker reflecting lipid accumulation or whether it has a functional role in regulating lipid metabolism. We focused our *in vitro* studies toward metabolic pathways that require bicarbonate. To further determine the role of Car3 in metabolism, we measured *de novo* fatty acid synthesis with *in vitro* radiolabeled experiments and examined metabolic biomarkers in Car3 knockout and wild type mice fed high-fat diet. Specifically, we analyzed body weight, body composition, metabolic rate, insulin resistance, serum and tissue triglycerides. Our results indicate that Car3 is not required for *de novo* lipogenesis, and Car3 knockout mice fed high-fat diet do not have significant differences in responses to various diets to wild type mice.

## Introduction

**Fig 1:**
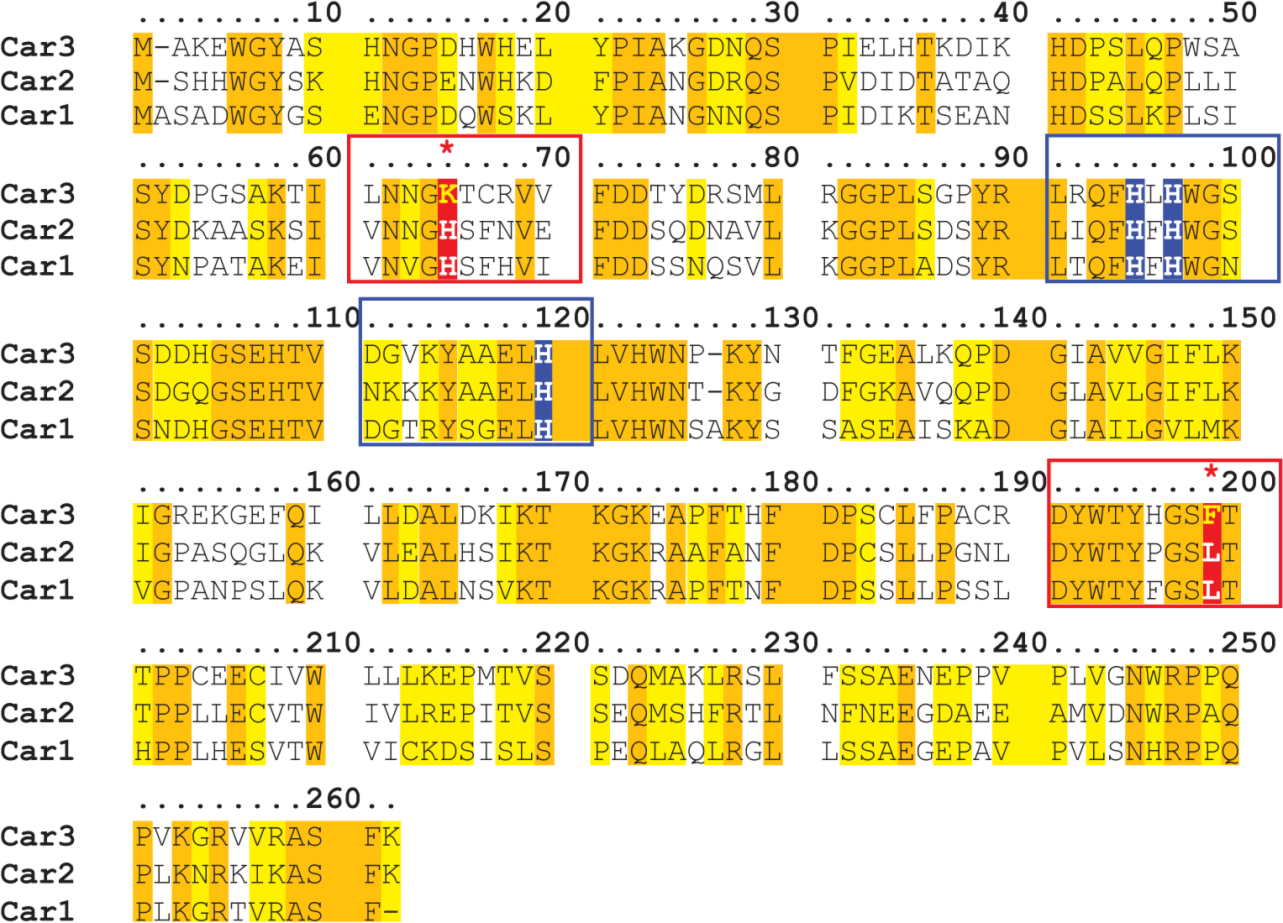
Amino acid sequence alignment of cytosolic carbonic anhydrases reveals key enzyme amino acids changes that affect activity. CA III (Car3) amino acid alignment with carbonic anhydrase family members Car1 and Car2. Car3 shares a 57%/56% identity and 74%/75% similarity with Car2 and Car1 respectively. Car3 has a 69% identity with either Car2 or Car1. Amino acids highlighted in yellow are identical between the three CAs. Amino acids highlighted in orange are shared between Car3 and either Car1 or Car2. The active site of all carbonic anhydrases contain a Zn^2+^ and three histidine residues (highlighted in blue) [12]. Two key amino acid differences (highlighted in red), at Lys64 and Phe198, are responsible for the substantially lower rate of regeneration of the active form for carbonic anhydrase 3 compared to the other two CAs [16, 17]. A lysine at amino acid 64 results in a less effective proton shuttle, [13, 15] and Phe198 causes a steric hindrance at the active site [14].

Carbonic anhydrases (CAs) are isozymes that catalyze the carboxylation of water into carbonic acid, which spontaneously dissociates into bicarbonate and protons (H_2_O + CO_2_ ←H_2_CO_3_→ HCO_3_^-^ + H^+^) [1, 2]. There are 16 unique isozymes in the vertebrate carbonic anhydrase gene family, or the α-CA family [3]. Carbonic anhydrases are involved in a wide variety of functions: respiration, acid-base homeostasis, ion transport, bone resorption, taste preferences, ureagenesis and gluconeogenesis [4–8]. The subcellular localization of these proteins is diverse: eight localize in the cytosol, five are transmembrane or membrane bound, two localize to the mitochondria, and one is secreted [3]. The α-CA family is comprised of active enzymes that contain a functional catalytic site (CA I, II, III, IV, VA, VB, VI, VII, IX, XII, XIII, XIV, XV) as well as catalytically dead enzymes known as carbonic anhydrase related proteins (CA-RP VIII, X and XI) [3]. While most of the active carbonic anhydrases have some of the highest activity rates in biology, CA III has 0.16% of the activity compared to CA II [9, 10]. Unlike the CA-RP proteins, which have mutated histidine (His) residues in the active catalytic site leaving them enzymatically inactive, CA III still maintains the histidine site for CAs, containing a zinc molecule surrounded by three His residues [3, 11, 12]. However, CA III does differ in amino acid alignment with the closely related CA I and CA II proteins at two important residues, as depicted in (Fig 1), lysine 64 (Lys) and phenylalanine 198 (Phe), which dramatically decrease the activity of CA III [13–17]. While the function of CA III is currently unknown it has been speculated to be involved in metabolism, oxidative damage response, and mitochondrial ATP synthesis [18–22].

CA III (Car3 in mice) is highly abundant in tissues that can store lipids in mice: liver, brown fat and white adipose tissues (approximately 24% of soluble protein in white adipose), and slow-twitch skeletal muscle [23]. Car3 appears to be nutritionally regulated; during starvation Car3 mRNA decreases in rodent livers [24–26] and Car3 mRNA subsequently increases upon refeeding. Car3 protein expression also increases in rodents fed Western-type high fat diets in both livers (31–83% increased protein) and white adipose tissue (97–129% increased mRNA) [27–29] Additionally, dietary stressors on rodents, including protein depletion and alcohol consumption, decrease the mRNA and protein levels of Car3 in the liver. [30–36]. Car3 expression also changes in response to insulin, a key metabolic hormone. Car3 mRNA expression increases in response to insulin in adipose tissue [18] and Car3 protein decreases in liver when insulin is decreased in rats through injection of streptozotocin [37, 38]. Since *de novo* lipogenesis occurs during nutrient-rich states when insulin levels are high [39], and there is the requirement of bicarbonate substrate in the committed step of fatty acid synthesis, acetyl-Coa → malonyl-CoA, [40], we hypothesized that carbonic anhydrase III is providing the necessary bicarbonate for *de novo* lipogenesis. CAs that are highly enzymatically active have been suggested to provide the bicarbonate for *de novo* lipogenesis; animals and cells with pharmacologically decreased CAs have decreased *de novo* lipogenesis [41–43], but the role of Car3, with much lower enzymatic activity, in *de novo* lipogenesis has not been previously studied.

In rodent models of diabetes/obesity, Car3 protein expression in liver and/or adipose tissue decreases in obese Zucker rats and in *ob/ob* mice compared to wild type rodents [23, 44, 45]. Even though Car3 may decrease, acetyl Co-A carboxylase (ACC) protein expression is up-regulated in adipose tissue of obese Zucker rats [44], perhaps contradictory with a role for Car3 in *de novo* lipogenesis.

The small molecule drug Niclosamide (Nicl) has recently been implicated as a potential therapeutic for type 2 diabetes (T2D) and fatty liver disease in mice fed HFD [46, 47]. Niclosamide prevents insulin resistance in high-fat diet fed mice, and improves insulin sensitivity in *db/db* mice, a leptin signaling deficient rodent model [47, 48]. Excess storage of lipids in liver, adipose, and muscle tissues contributes to T2D, and mice treated with niclosamide have reduced fat accumulation in liver and decreased liver triglycerides in a fatty liver mouse model [47]. In this study, we used two-dimensional differential gel electrophoresis (2D-DIGE) to identify Car3 as a protein highly expressed in DIO mice with fatty liver, but down-regulated in DIO mice treated with niclosamide. This result suggests that Car3 may reflect the lipid content of tissues.

To determine whether Car3 has a functional role in lipid content we characterized the previously described *Car3 -/-* mice [49]. No obvious deleterious phenotypes have been reported in previous studies on Car3 knockout mice; however, the effect of specialized diets on these knockout mice was not previously researched [19, 20, 22, 49, 50]. In this study, we investigate Car3 function in *de novo* lipogenesis by measuring fatty acid synthesis in isolated mouse hepatocytes, and during induced obesity through high-fat diet by measuring subsequent changes in body weight, dyslipidemia, insulin resistance, food intake, and energy expenditure.

## Materials and methods

### 2D-DIGE protocol and western blot confirmation

#### Animal care

20-week old diet-induced obesity (DIO) mice were purchased from Jackson Labs, with mice starting on the high-fat diet (D12492, Research Diets) at 6 weeks. At 20 weeks of age, mice were treated with 10mg/kg niclosamide or DMSO vehicle control for 12 days by daily intraperitoneal (IP) injections. Mice were harvested to obtain liver tissue. Mice were maintained at the University of North Carolina (UNC) under protocols specifically approved for this study by the UNC Institutional Animal Care and Use Committee (IACUC).

#### 2D-Differential gel electrophoresis (2D-DIGE) of niclosamide-treated mouse liver

Liver lysates were prepared, and 2D-DIGE experiments performed by the University of North Carolina Systems-Proteomics Center core facility as previously described [51, 52]. Protein spots were identified using peptide mass fingerprinting tandem mass spectrometry data by the Yale Mass Spectometry and Proteomics Resource Core (New Haven, CT), according to previously described methods [51–53].

#### Western blotting of mouse liver lysates

20 week old DIO high-fat diet fed mice were treated for 12 days with either 5 mg/kg, 25 mg/kg niclosamide, or DMSO (vehicle) by daily IP injections (40 μl). On the last day of treatment, mice were sacrificed and livers harvested and frozen on dry ice. For western blot analysis, liver samples were homogenized and sonicated in 10 volumes of RIA lysis buffer (50 mM Tris-Cl pH 7.5, 150 mM NaCl, 1% Triton X-100, 0.1% SDS, 0.5% sodium deoxycholate, 1 mM PMSF, 1 mM beta-glycerolphosphate, 2.5 mM sodium pyrophosphate, 1 mM sodium orthovanadate, 1X Sigma-Aldrich P8340 protease inhibitor cocktail). Homogenates were clarified by centrifugation at 16,000xg for 10 minutes at 4°C, and protein in the supernatant was quantified using the Bio-Rad Dc Assay (Hercules, CA). Protein samples in loading buffer (Life Technologies NP0007) were loaded at 50 μg per well on 4–12% NuPAGE gels and separated for 45 minutes at 200V, then transferred to Immobilon-FL blotting membrane at 300 mA for 1 hour. Blots were blocked with 5% milk in TBS, then probed for carbonic anhydrase III (Santa Cruz Biotechnology, sc-50715) and subsequently actin (MP Biomedicals, #69100) to control for total protein loaded. Secondary antibodies were (IRDye infrared antibodies, LI-COR Biosciences, Lincoln, NE) [51]. The Odyssey Infrared Imaging System was used for quantification of the relative intensity ratio of Car3/Actin (LI-COR Biosciences, Lincoln, NE).

### Animal care for Car3 knockout mice

*Car3* -/- mice were obtained from NIH (kind gift from Dr. Levine) on a mixed 129SvEv background as previously generated [49]. To outcross the *Car3* knockout alleles into a C57BL/6J background more appropriate for metabolic studies [54–56], male progeny were backcrossed with C57BL/6J female at least 8 generations. Sibling matched males were used in all analyses, unless otherwise specified. Mice were weaned at 3–4 weeks of age and placed on standard chow (2020X, Teklad Diets, Madison WI). Mice were maintained at the University of North Carolina under protocols specifically approved for this study by the UNC IACUC.

### Hepatocyte isolation and *de novo* lipogenesis assays

Hepatocytes were isolated using a protocol adapted from Wendel et al. [57]. Littermate female mice fed standard chow were anesthetized by IP injection of 250mg/kg tribromoethanol. Once mouse toes were unresponsive to touch, a 24G catheter was inserted into the portal vein. The liver was perfused with Perfusion Buffer 1 (115mM NaCl, 25mM HEPES, 5mM KCl, 1mM KH_2_PO_4_, 525μM EGTA, pH 7.4) warmed to 37°C, and then Perfusion Buffer 2 (115mM NaCl, 25mM HEPES, 5mM KCl, 1mM KH_2_PO_4_, 2.5mM MgSO_4_, 1.6mM CaCl_2_, 1.4–1.8mg/ml collagenase Type I (Worthington Chemicals, Lakewood, NJ), pH 7.4) warmed to 37°C. After perfusion was complete, the liver was excised and hepatocytes were released by gently tearing the liver lobes in chilled Isolation Medium, DMEM (Gibco #31053–028), 5% Fetal Bovine Serum, 1% Penicillin/Streptomycin, 2mM L-Glutamine, 1x MEM Non-Essential Amino Acids, 4μg/ml insulin, 0.1μM Dexamethasone. The hepatocytes were then filtered through a 100μm cell strainer and centrifuged at 50 x *g* for 2 min at 4°C. The cells were washed twice with cold Isolation Medium, and resuspended in warmed Isolation Medium for cell counting and plating. Hepatocytes were plated on collagen-coated 6-well dishes with 1x10^6^ cells per well, and incubated at 37°C with 5% CO_2_. After cells attached to the plates (4 h post-plating), media were changed to warmed L-15 media with 1% penicillin/streptomycin. Sixteen to 20 h after plating, hepatocytes were rinsed with warmed 1x PBS, (Gibco #14190–144) and then treated with 2ml of Assay Medium, L15 media (Gibco #11415–064), 1% penicillin/streptomycin, 0.8μCi/ml [1,2-^14^C] acetic acid (Perkin Elmer Life Sciences, NEC553001MC) for 2 hours. The hepatocytes were rinsed with warmed 1x PBS, and then lysed with methanol and scraped. Lipids were extracted from hepatocyte lysates using water:chloroform:methanol (0.8:2:2 v/v/v). Lipids from the chloroform layer were collected, dried down, and counted in scintillation vials. Protein quantifications were made using duplicate plates from the same hepatocyte isolation. Cells were scraped and sonicated in SDS lysis buffer (2% SDS, 60mM Tris-Cl pH 6.8, 1mM PMSF, 2.5 mM sodium pyrophosphate, 1mM beta-glycerolphosphate, 1mM sodium orthovanadate, 1X Sigma-Aldrich P8340 protease inhibitor cocktail). Cell lysates were clarified by centrifugation at 16,000xg for 10 minutes at 4°C, and protein in the supernatant was quantified using the Bio-Rad Dc Assay (Hercules, CA).

### Metabolic characterization of mice on high fat diet

At 5–6 weeks of age, mice either remained on standard chow or were placed on a high-fat diet [54, 58, 59]. The standard chow caloric consumption is 24 kcal% protein, 16 kcal% fat, and 60 kcal% carbohydrate, and the energy density is 3.1 kcal/g food (2020X, Teklad Diets, Madison WI). The high-fat diet caloric consumption is 20 kcal% protein, 60 kcal% fat, and 20 kcal% carbohydrate, and the energy density is 5.24 kcal/g food (D12492, Research Diets, New Brunswick, NJ).

#### Metabolic chambers and body composition

Mouse body composition, energy expenditure, activity, and food intake were measured at the Animal Metabolism Phenotyping Core at UNC under the supervision of Dr Kunjie Hua. Body composition was determined by MRI at 11 weeks of age (EchoMRI, Houston, TX) [60]. Energy expenditure, activity, and food intake were measured at 13–14 weeks of age using CaloCages (PhenoMaster, TSE systems, Chesterfield, MO). Data were collected for 2 days; the first day was considered the acclimation period and was excluded from data analysis [61]. Mice had unlimited access to food and water for the entire duration in metabolic chambers.

#### Serum measurements

Fasted mice were starved overnight (15–16 hours) [62] and measurements were taken at the beginning of the light cycle. To better synchronize the feeding schedule for fed measurements, food was removed from the cages during the light cycle, and mice were fed *ad libitum* during the dark cycle. Fed measurements were taken at the beginning of the light cycle after three nights of entrainment as described [63]. Glucose was measured with a drop of whole blood using a glucometer (Bayer Contour, Leverkusen, Germany). Whole blood was collected from mice using Microvette® tubes (CB300Z, Sarstedt, Nümbrecht, Germany) under fasting and fed conditions. To extract serum from whole blood, the blood was allowed to clot for 30–90 min at room temperature and then centrifuged at 2000 x *g* for 20 min (4°C), per provided instructions from Microvette. Insulin levels were determined using a mouse insulin ELISA kit (Crystal Chem Inc, Downers Grove, IL). Serum triglycerides (Wako Diagnostics, Richmond, VA), non-esterfied fatty acids (Wako Diagnostics, Richmond, VA), cholesterol (Stanbio, Boerne, TX) and glycerol (Sigma-Aldrich, St. Louis, MO) were determined with enzymatic colorimetric assays.

#### Oral glucose tolerance test (GTT)

Sixteen-week-old mice were fasted overnight for 14–15 hours followed by oral gavage of 2 mg/g body weight of glucose. Blood glucose was measured at 0, 15, 30, 60, and 120 minute time points using a glucometer, as described previously described [64, 65].

#### Tissue triglycerides

At 18 weeks of age, mice were sacrificed and tissues were weighed and collected for further analysis. Liver triglycerides were measured from the left lobe, while muscle triglycerides were measured from calf muscle using a protocol adapted from [66]. Tissues were homogenized on ice using the BeadBug (Benchmark Scientific, Edison, NJ) with 50–100 mg of tissue in 10 or 20 volumes of 5% NP-40 in water (10 volumes for liver samples and 30 volumes for muscle samples). Samples were boiled @ 100°C for 5 min, then cooled to room temperature twice. The samples were then centrifuged at 16,000 x *g* for 2 min to pellet denatured DNA and proteins. Supernatants were then used to measure triglycerides using a colorimetric assay (Sigma-Aldrich, St. Louis, MO).

#### Body temperature and thermoregulation

Body temperatures from mice fed standard or high-fat diet were measured by rectal probe under fasting (15–18 hours) and fed (*ad libitum*) conditions (MicroTherma 2, ThermoWorks, American Fork, UT) [67]. Adult female mice, 8–13 weeks of age, were fasted overnight and exposed to 4°C for 5 hours [68].

### Statistics

All data and statistics were analyzed using GraphPad Prism 7. Data are expressed as mean ± SD. Significance was determined using Tukey’s multiple comparisons for all figures except for body weights, energy expenditures, RER, GTT area under the curve (AUC), and tissue triglycerides. For GTT AUC and tissue triglycerides, significance was determined using unpaired T-test. A significance threshold of P < 0.05 was applied. For body weight, energy expenditure, and RER, discovery was determined by multiple T-tests using the two-stage linear step-up procedure of Benjamini, Krieger and Yekutieli, with Q = 1% [69]. Each row was analyzed individually, without assuming consistent SD.

## Results

### Niclosamide treated mice have lower Car3 protein expression

**Fig 2:**
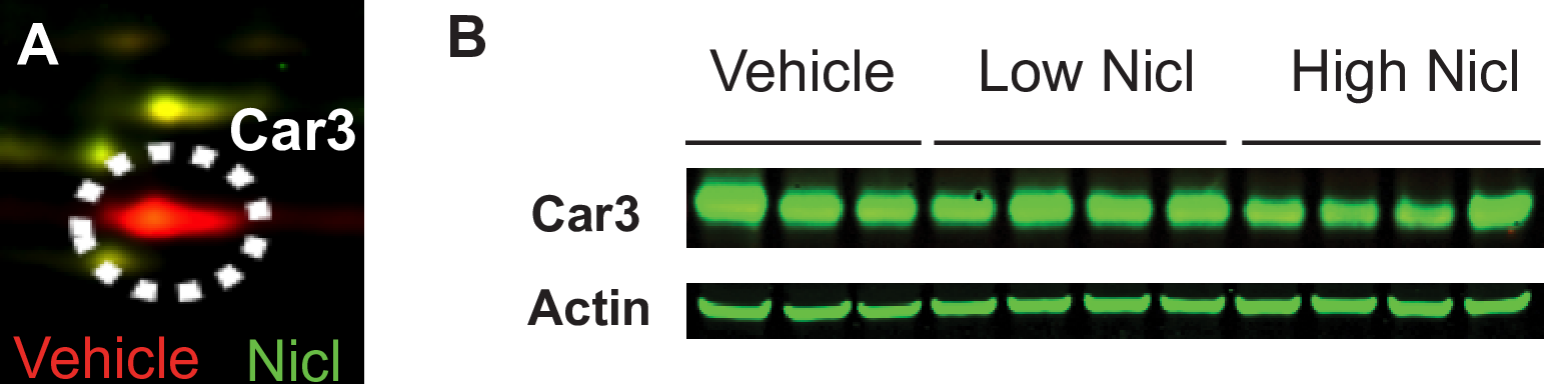
2D-DIGE identifies Car3 as a protein with decreased expression in livers of high-fat diet fed mice treated with Niclosamide. (A) 2D-DIGE of liver lysates from 20 week-old diet-induced obesity (DIO) mice treated with 10mg/kg niclosamide (green) or vehicle (red) for 12 days. Car3 protein is highlighted in the white circle. (B) Independent validation by Western blot analysis of Car3 from DIO mice treated with vehicle, low niclosamide (5mg/kg), or high niclosamide (25mg/kg) for 12 days.

In previous unpublished experiments, we observed that mice with fatty liver induced by diet-induced obesity (DIO) and treated with niclosamide had lower liver triglycerides and improved insulin sensitivity compared to untreated mice, which has been independently confirmed [47]. In an effort to identify which proteins might change in response to niclosamide treatment, we used 2D-DIGE analyses to compare liver protein expression from high-fat DIO treated with niclosamide or vehicle. Spots were then analyzed for differences in expression. One of the most abundant differentially expressed spot on the 2D-DIGE gel was excised and identified as Car3 (Fig 2A). In order to verify that Car3 protein expression changed with niclosamide treatment, we performed western blots on liver lysates from additional DIO mice treated with vehicle, 5 mg/kg or 25 mg/kg body weight doses of niclosamide (Fig 2B). The western blots confirmed the 2D-DIGE results with high and low doses of niclosamide leading to lowered Car3 protein levels (standardized to actin) compared to the vehicle-treated mice (mean relative Car3 intensities of 1.56, 2.16 and 2.54 respectively).

### Car3 is not required for *de novo* fatty acid synthesis

**Fig 3:**
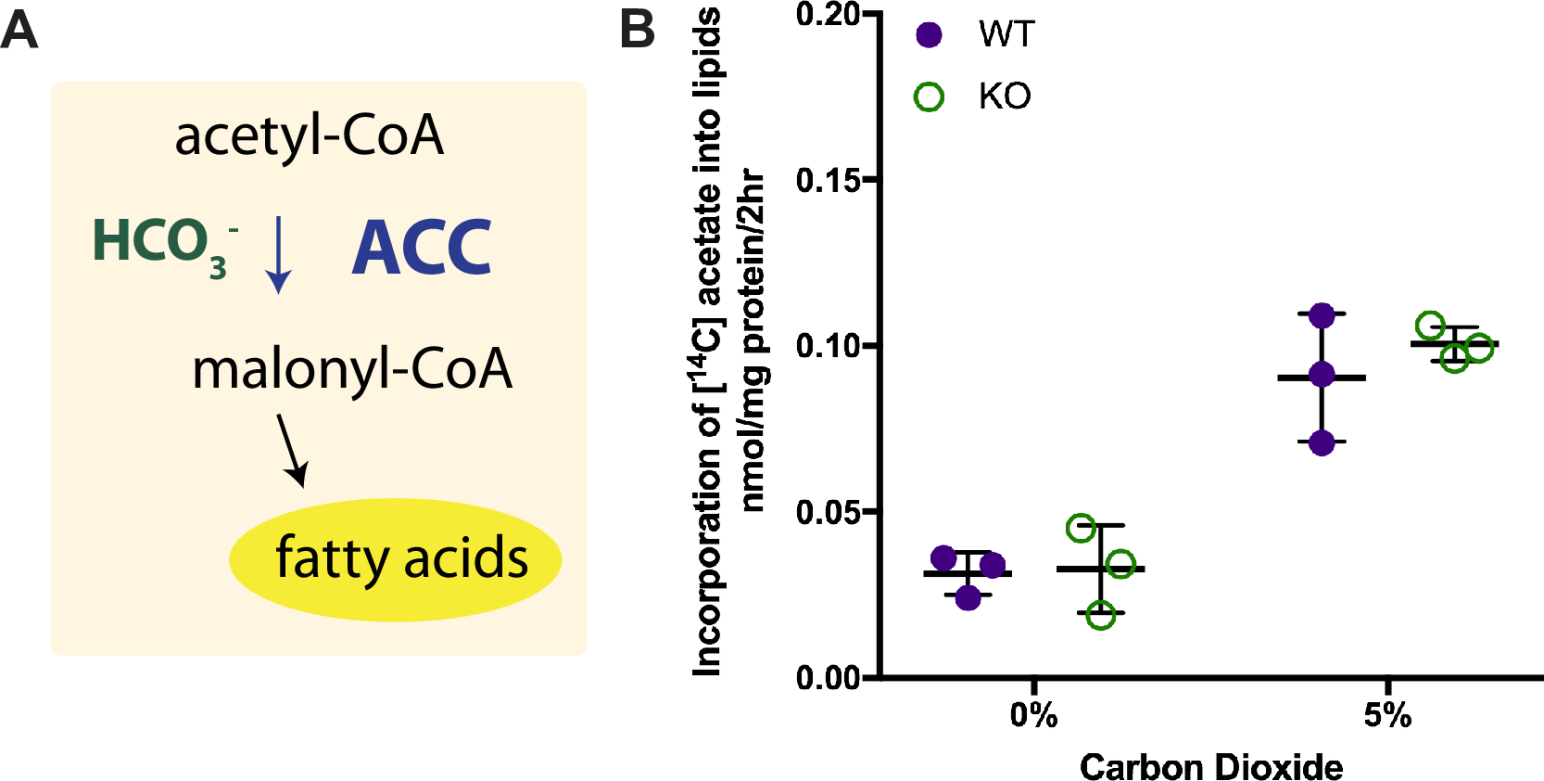
Car3 is not required for de novo lipogenesis. (A) The rate-limiting step in fatty acid synthesis requires bicarbonate for the carboxylation of acetyl-CoA to malonyl-CoA by acetyl-CoA carboxylase (ACC). (B) Wild type (WT) and *Car3 -/-* (KO) hepatocytes show no difference in *de novo* lipogenesis. Hepatocytes were labeled with [1,2-^14^C] acetate in the presence and absence of carbon dioxide in bicarbonate free media. Data shown are from three experiments performed with six replicates.

Given the correlation between Car3 expression and fatty liver we wondered whether Car3 plays a role in lipid homeostasis. Given that bicarbonate is required for lipid synthesis, we tested the hypothesis that Car3 might be required for lipogenesis, using the radiolabeled-acetate incorporation assay to compare *de novo* fatty acid synthesis of wild type and *Car3-/-* primary mouse hepatocytes. Because the rate-limiting step of fatty acid synthesis requires bicarbonate (Fig 3A), we were concerned that traditional cell culture incubations utilizing a bicarbonate-based buffering system would mask any potential differences in [1,2-^14^C]acetate incorporation into lipid. To account for this, we used L-15 media, a cell culture media with a bicarbonate-free buffering system, to culture and assay the hepatocytes. We also performed the assay in the presence and absence of carbon dioxide during incubation. For both with or without CO_2_, the incorporation of [1,2-^14^C]acetate into lipids was statistically indistinguishable between wild type and *Car3 -/-* cells. (Fig 3B) The incorporation of [^14^C]acetate into fatty acids was 65–67% higher in the hepatocytes incubated with 5% CO_2_, as expected, since that increases free bicarbonate in the media available for the first step in fatty acid synthesis (Fig 3A). These results indicate that Car3 expression has no significant effect on *de novo* lipogenesis in primary liver cells. Liver is the primary site of *de novo* lipogenesis in mammals.

### Weight gain, body composition, and chow consumption is not affected by Car3

**Fig 4:**
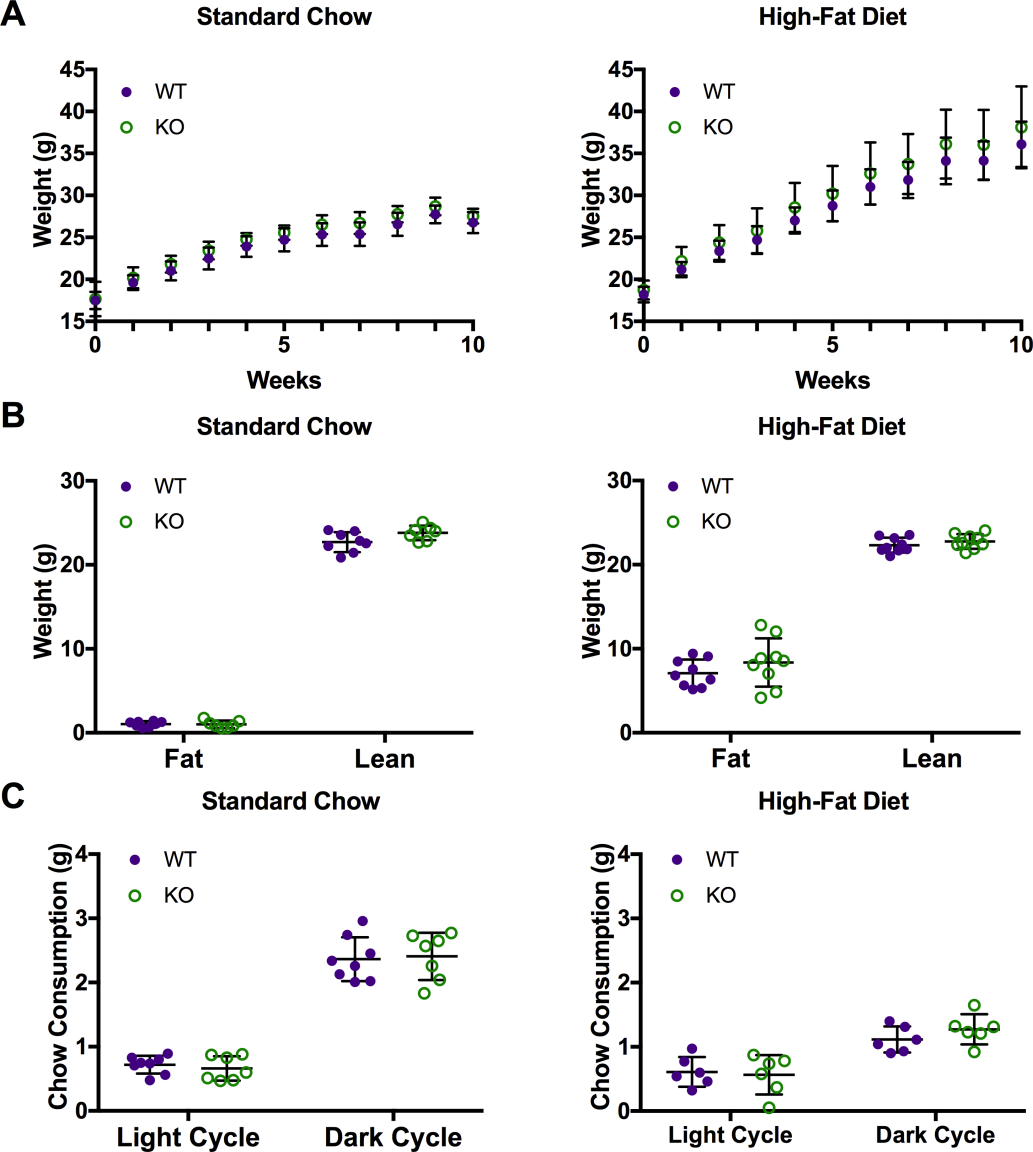
*Car3 -/-* mice fed high-fat diet showed no difference in weight gain, food consumption, or body composition. (A) Weight gain of wild type (WT) and *Car3 -/-* (KO) mice fed normal chow (n = 7–8) or high-fat diet (n = 9) starting at 5 weeks of age. (B) Body composition, fat and lean mass, of wild type and *Car3 -/-* mice measured at 11 weeks old by MRI. (C) Chow consumption of mice fed standard chow (n = 7–8) or high-fat diet (n = 6) during light and dark cycle measured at 13–14 weeks old.

To determine if Car3 affects body weight or composition, wild type and *Car3 -/-* mice were put on a high-fat diet (HFD) or standard chow at 5 weeks of age and measured once a week for 10 weeks. Body weights of wild type and *Car3 -/-* mice did not differ significantly at any age for either standard chow or high-fat diet (Fig 4A). After 8–9 weeks on the diets, food consumption was monitored over a pre-acclimated 24-hour period in a metabolic chamber (Fig 4B). For both diets, Car3 did not affect food consumption. Wild type and *Car3 -/-* mice ate the same amount of standard chow or HFD during both the light and dark cycle. To see if fat and lean mass were individually affected, we measured body composition on standard chow and high-fat diet. The lean masses of wild type and *Car3 -/-* mice were not statistically different, with the overall mean at 22.85 ± 1.058g (Fig 4C). Similarly, fat masses of wild type and *Car3 -/-* mice were not significantly different, with overall mean fat mass on standard chow at 1.031 ± 0.3721 and on high-fat diet at 7.729 ± 2.358 (Fig 4C). Mice on HFD had an increased contribution of fat mass compared to the mice on standard chow, as expected. These results suggest that Car3 is not affecting weight gain or fat and lean mass composition under ad libitum feeding conditions.

### Car3 has no significant effect on total energy expenditure or energy substrate utilization with standard chow or HFD fed mice

**Fig 5:**
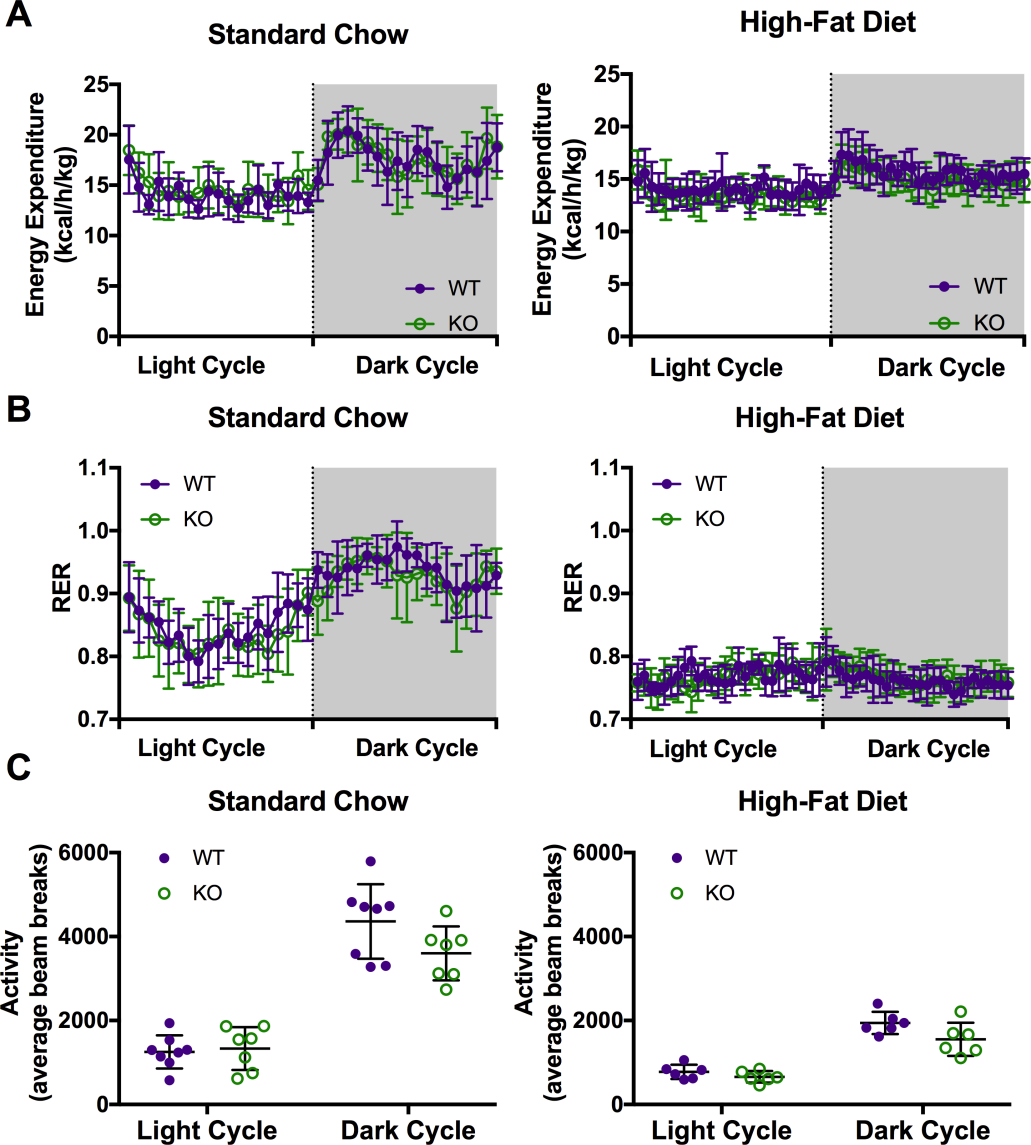
*Car3 -/-* mice show no changes in total energy expenditure, RER, or activity. Wild type (WT) and *Car3 -/-* (KO) energy expenditure (A), Respiratory Exchange Ratio (B), and activity (C) measured over a 24 hour period in 13-14-week-old mice on a standard chow (n = 7–8) or high-fat diet (n = 6). Standard chow fed to mice at weaning (3 weeks of age), mice fed high-fat diet were switched to HFD at 5 weeks of age.

To determine if Car3 affects fuel source utilization, we measured total energy expenditure, energy substrate usage, and physical movement activity in metabolic chambers. Wild type and *Car3 -/-* mice on both diets showed no difference in activity or energy expenditure (Fig 5A). To measure contribution of lipids or carbohydrates to energy usage, respiratory exchange ratios (RER) were calculated for the wild type and *Car3 -/-* mice fed standard chow and HFD; an RER equal to 0.7 indicating fatty acid oxidation is the main source of energy, whereas an RER equal to 1.0 or greater indicates carbohydrates are the primary fuel source. Wild type and *Car3 -/-* mice have similar RER values throughout the 24-hour monitoring period, and the light and dark cycle values were not statistically different for either standard chow or HFD-fed mice (Fig 5B). During both the light and dark cycle, there were also no significant differences in movement activity/average beam breaks for wild type and *Car3 -/-* mice fed standard chow or HFD (Fig 5C).

### Car3 has no effect on HFD-induced insulin resistance

**Fig 6:**
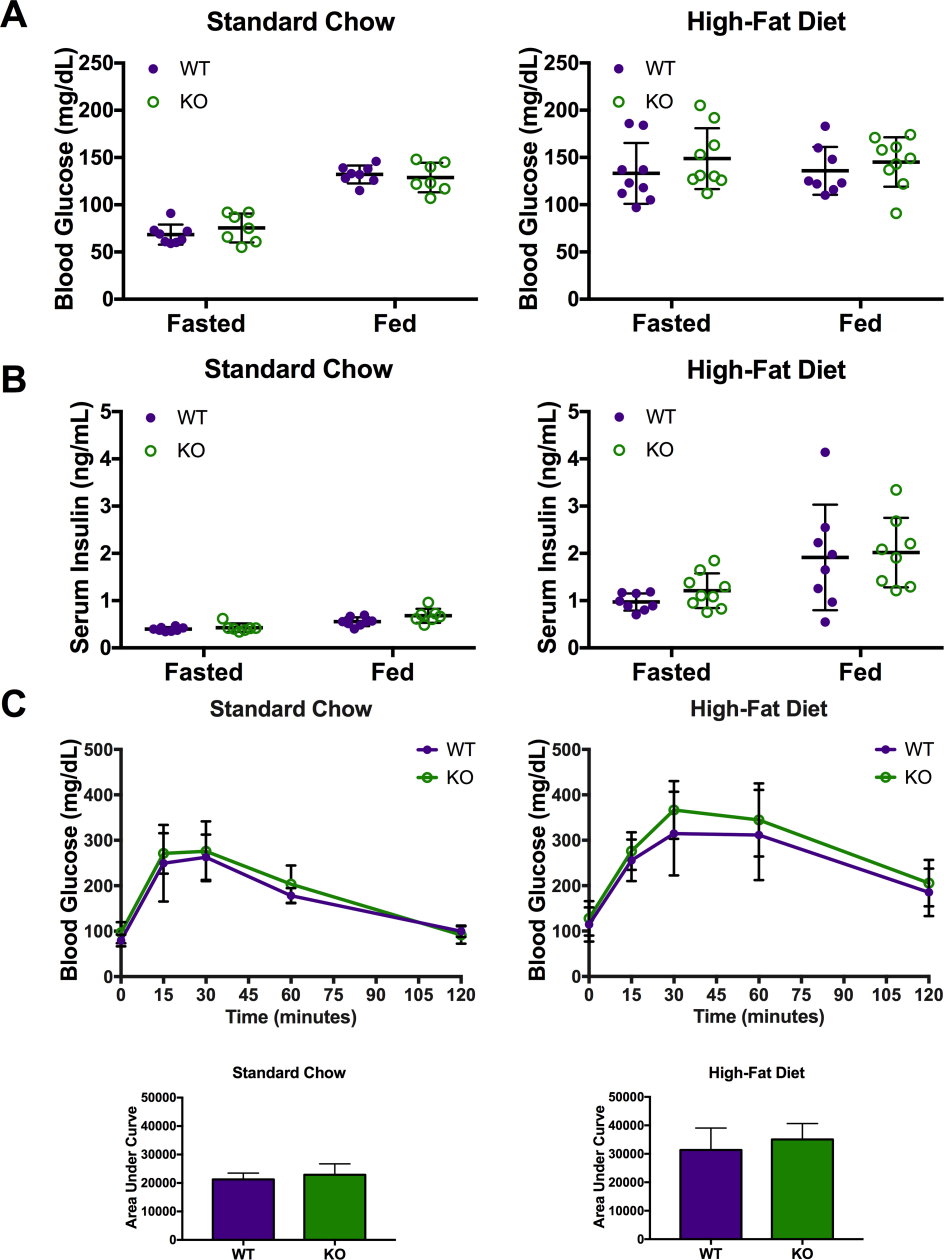
*Car3 -/-* does not affect high-fat diet-induced insulin-resistance. Fasting and fed blood glucose (A) and serum insulin (B) from wild type (WT) and *Car3 -/-* (KO) mice fed standard chow (n = 7–8) or high-fat diet (n = 8–9) for 10 weeks. (C) Oral glucose tolerance test (with area under the curve below) performed on standard chow (n = 7–8) and high-fat diet (n = 4–5) mice after 11 weeks on the respective diet regime.

Mice fed high-fat diets can have elevated fasting blood glucose and insulin reflecting lower insulin sensitivity [58, 70]. To test the effect of Car3 on insulin sensitivity, we performed glucose tolerance tests (GTT) and collected fasting blood glucose and insulin on mice fed either standard chow or high-fat diet. Since liver Car3 mRNA expression is highest in the fed state, to maximize possible differences between wild type and knockout mice, we also obtained fed blood measurements [25]. Both wild type and *Car3 -/-* mice fed standard chow had lower blood glucose and insulin in the fasting state than in the fed state (as expected); similarly HFD-fed mice had elevated fasting blood glucose and insulin compared to standard chow fed mice (Fig 6A and 6B), as also expected. Glucose tolerance tests in mice fed standard chow and high-fat diet were not statistically significant between wild type and *Car3 -/-* mice in individual glucose measurements or area under the curve calculations (Fig 6C).

### Car3 knockouts have normal serum and tissue lipid/fatty acid biomarker levels

**Fig 7:**
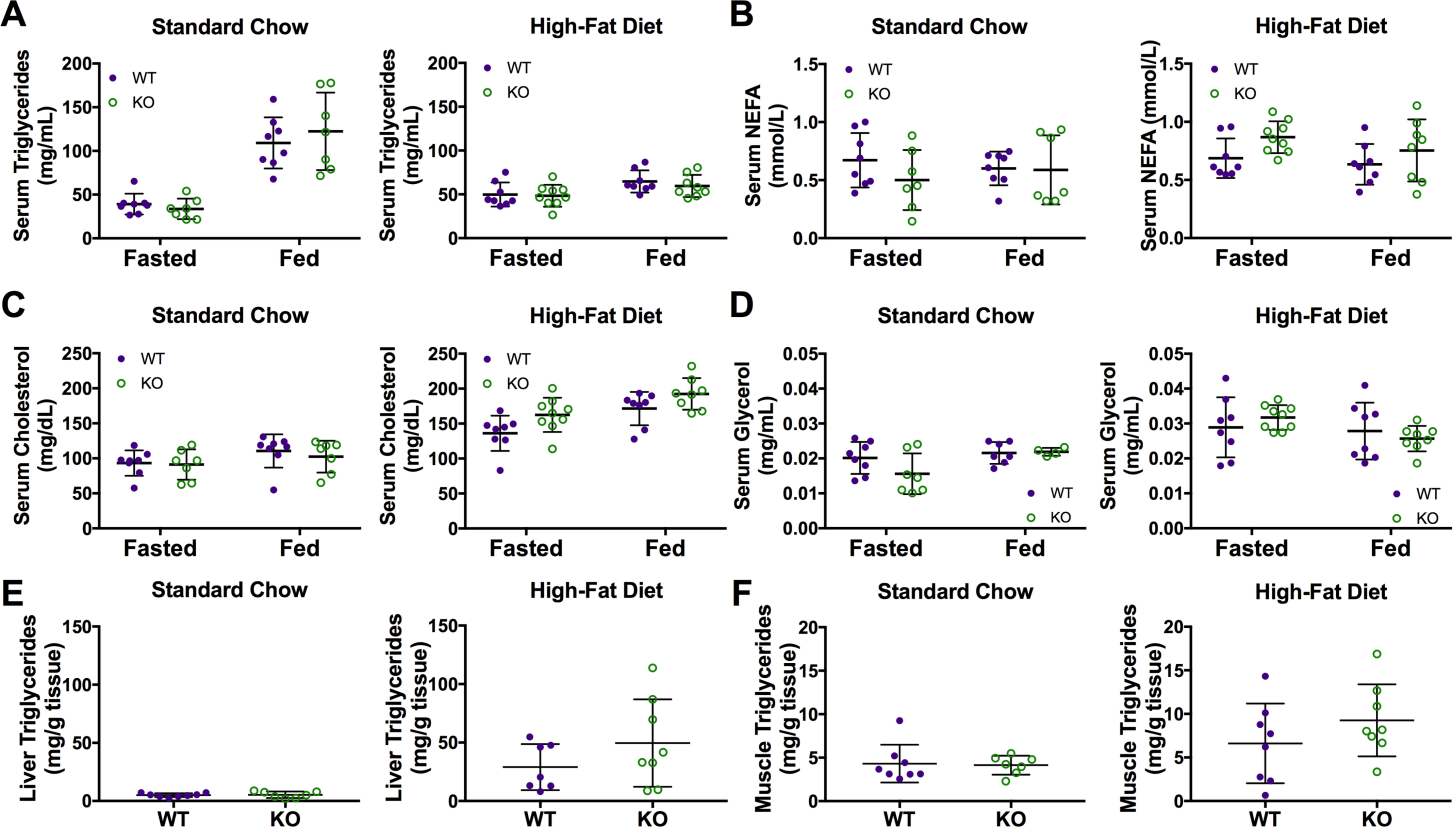
*Car3 -/-* mice show no difference in serum and tissue lipids and lipid substrates. Fasting and fed serum triglycerides (A), non-esterfied fatty acids (B), cholesterol (C), and glycerol (D) of wild type (WT) and *Car3 -/-* (KO) mice fed standard chow (n = 7–8) or high-fat diet (n = 8–9) for 10 weeks. Liver (E) and muscle (F) triglyceride content in mice fed standard chow (n = 7–8) or high-fat diet (n = 8) for 13 weeks.

To see if Car3 plays a role in increased liver triglycerides found in mice with fatty liver, we measured serum and tissue lipids (fatty acids and triglycerides). As stated previously, fed serum measurements were also obtained due to Car3 mRNA expression being highest in the liver in the fed state. Fasted and fed serum triglycerides, non-esterfied fatty acids (NEFA), cholesterol, and glycerol levels were all measured and found not statistically different between wild type and *Car3 -/-* mice on either diet (Fig 7A–7D). Liver and muscle triglyceride content also showed no difference between wild type and *Car3 -/-* mice (Fig 7E and 7F).

### Is Car3 required for lipid uptake/mobilization during thermogenesis?

**Fig 8:**
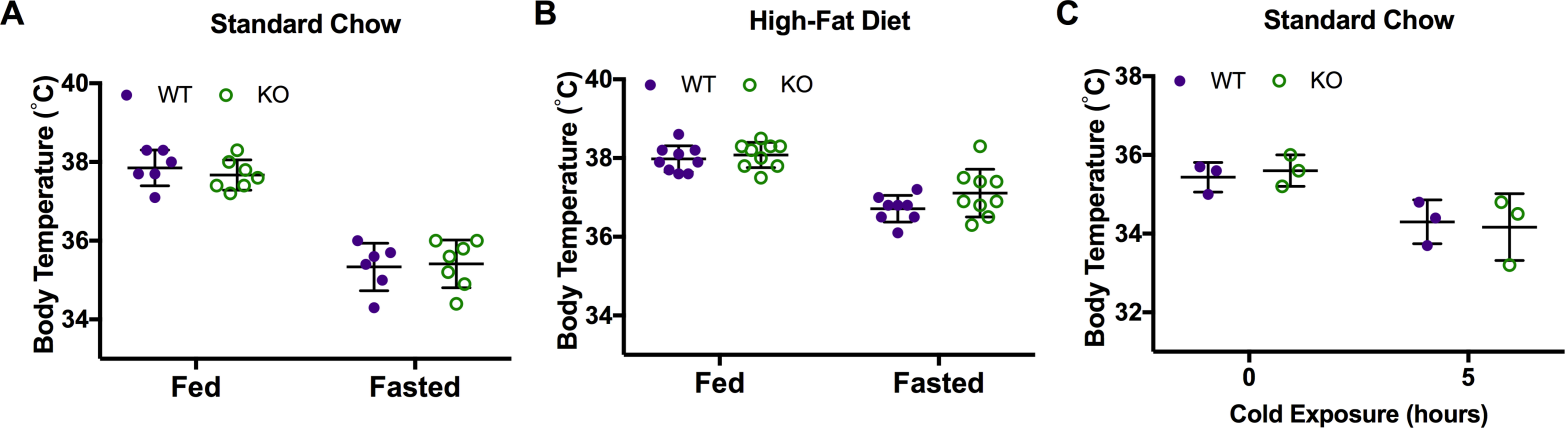
*Car3 -/-* mice have normal thermoregulation. Wild type (WT) and knockout (KO) body temperatures of adult mice aged 8–15 weeks at different states of thermoregulation. Fasted state is an overnight fast for 15–18 hours. (A) Females fed standard chow (n = 6–7). (B) Males fed HFD for 10 weeks, starting at age 5 weeks (n = 8–9) (C) Females were fasted overnight and subjected to 4°C for 5 hours (n = 3).

Given the abundance of Car3 in adipose and skeletal muscle and increase in fatty livers, this was somewhat reminiscent of the expression of CD36 protein, which also increases in fatty liver and is abundant in adipose tissue [71, 72]. One of the most profound phenotypes of CD36 knockout mice is the inability to maintain core body temperature under cold challenge due to suspected inability to uptake lipid and subsequently hydrolyze it for energy/heat, particularly in brown fat [73]. Given the similarity in expression of CD36 and Car3 proteins in lipid-storing tissues, we subjected *Car3 -/-* mice to cold temperature challenge. *Car3 -/-* mice are not impaired in their ability to maintain core body temperature in the cold (Fig 8).

## Discussion

Despite the abundance of Car3 in key metabolic tissues including white and brown fat, liver, and skeletal muscle its function remains unknown. The relatively high abundance of Car3 these tissues–in fats Car3 is nearly 30% of soluble protein and 8% in slow-twitch muscle [23, 49]–led to extensive analysis of *Car3 -/-* muscle function but showed relatively mild muscle phenotypes with normal “growth, development and life span” [49].

Although the exact function of Car3 remains unknown, many researchers postulate Car3 function is not as a carbon dioxide hydratase given its relatively miniscule enzymatic activity compared to Car1 and Car2 (0.16% with purified protein *in vitro* [10]). Additional evidences suggesting Car3 may not function to produce bicarbonate *in vivo* are observations that the most potent Car3 inhibitor is carbonate ion (10μM K_I_), which is in equilibrium with bicarbonate itself [10]. These investigators stated “it is difficult to explain why Nature preserved … such a “bad” catalyst” that is also most potently inhibited by a proposed equilibrium product.

Even though evidence suggests Car3 may have no functional role in producing bicarbonate, we still explored potential roles for Car3 in lipid homeostasis because Car3 protein is highly enriched in tissues that can accumulate or synthesize lipid, and because bicarbonate is essential for fatty acid synthesis [18]. The three prior studies characterizing Car3 knockout mice did not investigate phenotypes related to lipid synthesis or storage nor did these studies feed high-fat diets to find any phenotypes [20, 49, 50].

Although Car3 may not drive bicarbonate production *in vivo*, no one previously used *Car3 -/-* mice to demonstrate that Car3 is not required for *de novo* lipogenesis *in vivo* (Fig 3). Given the abundance of Car3 in lipid containing tissues, we decided to focus on the response of *Car3 -/-* mice fed a high-fat diet known to alter lipid homeostasis. We measured key metabolic indicators including: weight gain, body composition, chow consumption, energy expenditure, insulin resistance, and dyslipidemia. *Car3* deficiency had no effect on the biomarkers we tested, suggesting that Car3 is not responsible for the synthesis of fat nor does it affect the mobilization of fat in these tissues.

We identified Car3 as a protein increased in the liver in a mouse model fed high-fat diet but lowered with niclosamide (Fig 2), a drug shown to improve fatty liver and T2D-like symptoms [47]. Since Car3 liver protein expression was higher in DIO mice fed HFD, and lower in niclosamide treated HFD-fed DIO mice, we hypothesized that Car3 was involved in the development of fatty liver in this disease model. We saw no difference in liver triglycerides of wild type and *Car3 -/-* mice fed HFD, which suggests that Car3 is not required for accumulation of fat in the liver. While Car3 could be a biomarker for fatty liver disease in DIO mice, it is also possible that the effect of decreased Car3 seen in niclosamide-treated mouse livers may simply reflect lower fat content in the tissue, without regulating its decrease.

The high abundance of Car3 in selected tissues and relative lack of enzymatic activity suggest perhaps Car3 plays a non-enzymatic role. There is evidence that Car3 acts as a protectant against oxidative damage; cells transfected with Car3 are protected from apoptosis induced by hydrogen peroxide [21]. Car3 can also undergo the posttranslational modification by S-glutathionylation at two cysteine residues, Cys186 and Cys181 not found in Car1/2 [74–76]. S-glutathionylation of proteins is an early response to oxidative damage [77]. S-glutathionylation of Car3 increases with increased oxidative damage that occurs naturally as in aging [78] or with muscle damage [22]. Car3 contains numerous other additional cysteine residues not found in Car1/2. The concept of Car3 as an abundant “scavenging” protein to protect against oxidative damage is becoming an area of more research focus [79].

Interestingly, Car3 protein expression is altered in SOD1, copper zinc super oxide dismutase 1, providing another connection to oxidative stress responses in tissues. In *SOD1 -/-* mouse knockouts Car3 protein was identified as an abundant oxidized protein in liver that decreases over time specifically in knockouts [80]. Similarly, in the *‘toxic milk’* mouse mutant, Car3 protein is also greatly reduced in the liver; Car3 was shown to bind copper ions in addition to zinc [81]. (Toxic milk protein is an ATP7B transporter that helps regulate copper levels in the body.) Thus Car3 seems to have physiological connections to both metallic ions and oxidative stress responses. While our study has not demonstrated a role for Car3 in lipid metabolism, future characterization of *Car3 -/-* mice for roles in oxidative stress responses or for functions related to binding metal ions in cells may be more fruitful.

## Conclusion

In conclusion, we attempted to determine the role of Car3 in lipid metabolism/homeostasis after we discovered Niclosamide decreased Car3 protein in the livers of HFD fed mice under similar conditions that improve fatty liver and insulin sensitivity/diabetic symptoms in mice [46, 47]. Since Niclosamide decreases liver triglycerides and improves insulin sensitivity, we hypothesized that perhaps Car3 had a functional role in promoting fatty acid synthesis or lipid accumulation. We determined using mouse genetics that Car3 is not required for *de novo* lipogenesis. Additionally, we found that Car3 knockout and wild type mice fed either a standard chow or a HFD are not statistically different in weight, body composition, metabolic rate, insulin sensitivity, serum lipids, or tissue triglycerides. While Car3 is an abundant protein in many key metabolic tissues, the physiological role of Car3 in these tissues still remains to be determined.
